# *Trim37*-deficient mice recapitulate several features of the multi-organ disorder Mulibrey nanism

**DOI:** 10.1242/bio.016246

**Published:** 2016-04-04

**Authors:** Kaisa M. Kettunen, Riitta Karikoski, Riikka H. Hämäläinen, Teija T. Toivonen, Vasily D. Antonenkov, Natalia Kulesskaya, Vootele Voikar, Maarit Hölttä-Vuori, Elina Ikonen, Kirsi Sainio, Anu Jalanko, Susann Karlberg, Niklas Karlberg, Marita Lipsanen-Nyman, Jorma Toppari, Matti Jauhiainen, J. Kalervo Hiltunen, Hannu Jalanko, Anna-Elina Lehesjoki

**Affiliations:** 1 Folkhälsan Institute of Genetics, FI-00290 Helsinki, Finland; 2 Research Programs Unit, Molecular Neurology, University of Helsinki, FI-00290 Helsinki, Finland; 3 Neuroscience Center, University of Helsinki, FI-00790 Helsinki, Finland; 4 Institute for Molecular Medicine Finland FIMM, University of Helsinki, FI-00290 Helsinki, Finland; 5 Department of Pathology, Central Hospital of Tavastia, FI-13530 Hämeenlinna, Finland; 6 Department of Neurobiology, A. I. Virtanen Institute for Molecular Sciences, University of Eastern Finland, KuopioFI-70211, Finland; 7 Biocenter Oulu andFaculty of Biochemistry and Molecular Medicine, University of Oulu, FI-90220 Oulu, Finland; 8 Department of Anatomy, Faculty of Medicine, University of Helsinki, FI-00290 Helsinki, Finland; 9 Minerva Foundation Institute for Medical Research, FI-00290 Helsinki, Finland; 10 Biochemistry and Developmental Biology, Institute of Biomedicine, University of Helsinki, FI-00290 Helsinki, Finland; 11 Genomics and Biomarkers Unit, National Institute for Health and Welfare, Biomedicum, FI-00290 Helsinki, Finland; 12 Department of Endocrinology, Children's Hospital, University of Helsinki and Helsinki University Hospital, FI-00290 Helsinki, Finland; 13 Departments of Physiology and Pediatrics, University of Turku, FI-20520 Turku, Finland; 14 Department of Nephrology and Transplantation, Children's Hospital, University of Helsinki and Helsinki University Hospital, FI-00290 Helsinki, Finland

**Keywords:** Mulibrey nanism, Infertility, Fatty liver, Cardiomyopathy, Tumorigenesis, Growth failure

## Abstract

Mulibrey nanism (MUL) is a rare autosomal recessive multi-organ disorder characterized by severe prenatal-onset growth failure, infertility, cardiopathy, risk for tumors, fatty liver, and type 2 diabetes. MUL is caused by loss-of-function mutations in *TRIM37*, which encodes an E3 ubiquitin ligase belonging to the tripartite motif (TRIM) protein family and having both peroxisomal and nuclear localization. We describe a congenic *Trim37* knock-out mouse (*Trim37^−/−^*) model for MUL. *Trim37^−/−^* mice were viable and had normal weight development until approximately 12 months of age, after which they started to manifest increasing problems in wellbeing and weight loss. Assessment of skeletal parameters with computer tomography revealed significantly smaller skull size, but no difference in the lengths of long bones in *Trim37^−/−^* mice as compared with wild-type. Both male and female *Trim37^−/−^* mice were infertile, the gonads showing germ cell aplasia, hilus and Leydig cell hyperplasia and accumulation of lipids in and around Leydig cells. Male *Trim37^−/−^* mice had elevated levels of follicle-stimulating and luteinizing hormones, but maintained normal levels of testosterone. Six-month-old *Trim37^−/−^* mice had elevated fasting blood glucose and low fasting serum insulin levels. At 1.5 years *Trim37^−/−^* mice showed non-compaction cardiomyopathy, hepatomegaly, fatty liver and various tumors. The amount and morphology of liver peroxisomes seemed normal in *Trim37^−/−^* mice. The most consistently seen phenotypes in *Trim37^−/−^* mice were infertility and the associated hormonal findings, whereas there was more variability in the other phenotypes observed. *Trim37^−/−^* mice recapitulate several features of the human MUL disease and thus provide a good model to study disease pathogenesis related to TRIM37 deficiency, including infertility, non-alcoholic fatty liver disease, cardiomyopathy and tumorigenesis.

## Abstract (precis)

**Summary:** A congenic *Trim37*-deficient mouse model recapitulates several features of the human disorder Mulibrey nanism, and thus provides a good model to study disease pathogenesis related to TRIM37 deficiency.

## INTRODUCTION

Mulibrey nanism (MUL) is a rare autosomal recessive multi-organ disorder (OMIM #253250) (Perheentupa et al., 1970, 1973). The main findings include severe prenatal-onset growth restriction, cardiac dysfunction due to pericardial and myocardial fibrosis and/or hypertrophy, infertility, increased risk for tumors, metabolic syndrome and type 2 diabetes (Eerola et al., 2007; Karlberg et al., 2004a,b, 2005, 2009a, 2011; Lipsanen-Nyman et al., 2003). Hepatomegaly and fatty liver are common findings (Karlberg et al., 2004a, 2005). Most of the patients have a large head relative to stature and characteristic craniofacial features, including a triangular face with high, broad forehead, low nasal bridge and scaphocephaly with occipitofrontal bossing (Karlberg et al., 2004a). A peculiar high-pitched voice is common in nearly all pre-pubertal children (Karlberg et al., 2004a). Radiological findings include slender long bones, J-shaped sella turcica, small thoracic cage and fibrous dysplasia of the long bones (Karlberg et al., 2004a). Yellowish dots in the ocular fundi, unequal dispersion of retinal pigment and hypopigmentation are common ocular findings (Karlberg et al., 2004a). Some patients present with mild muscular hypotonicity in childhood and psychomotor development may be delayed, but no major neurological symptoms are evident (Karlberg et al., 2004a).

Loss-of-function mutations in the tripartite motif-containing 37 (*TRIM37*) gene underlies MUL with twenty-three different disease-associated mutations known to date [Avela et al., 2000; Doganc et al., 2007; Hämäläinen et al., 2004, 2006; Jagiello et al., 2003; Kallijärvi et al., 2005; Kumpf et al., 2013; the Leiden Open Variation Database (LOVD; http://databases.lovd.nl/shared/genes/TRIM37)]. *TRIM37* encodes a 108-kDa member of the tripartite motif (TRIM) protein family that comprises a RING finger, a B-box motif and a coiled coil domain (Kallijärvi et al., 2005). TRIM37 has been shown to possess E3 ubiquitin ligase activity and to have both peroxisomal and nuclear localization (Kallijärvi et al., 2002, 2005, 2006). It has, thus, been proposed to be involved in the peroxisomal functions of the cell (Kallijärvi et al., 2002, 2005). During mouse embryogenesis, TRIM37 is widely expressed in the epithelial-mesenchymal tissues, and in adult mice TRIM37 immunoreactivity is detected in many neural crest derived tissues. Especially abundant staining is seen in certain endocrine tissues and in the gonads (Kallijärvi et al., 2006). In addition to granular cytoplasmic TRIM37 staining, nuclear staining is detected in several tissues (Kallijärvi et al., 2006). Recently, following a high-content genome-wide siRNA-based screen to identify genes involved in regulation of centriole formation in human cells, TRIM37 was reported to have a role in preventing centriole reduplication events (Balestra et al., 2013). Moreover, it has been implicated as an oncogenic histone H2A ubiquitin ligase in breast cancer (Bhatnagar et al., 2014). However, the physiologic function of TRIM37 and the disease mechanisms underlying MUL are still unknown.

The mouse *Trim37* gene is highly similar to human *TRIM37*, with 88% identity on mRNA and 92% identity on protein level. We here describe the generation and detailed characterization of the congenic *Trim37* knock-out (*Trim37^−/−^*) mouse strain, which recapitulates several key features of MUL, thus providing a good model for studying disease mechanisms associated with TRIM37 deficiency.

## RESULTS

### Generation and phenotype of *Trim37^−/−^* mice

The *Trim37* knock-out (*Trim37^Gt(bgeo)FH^*, denoted here as *Trim37^−/−^*) mouse strain containing a >16 kb β-geo insertion vector in intron 2 of the *Trim37* gene (see Fig. S1A,B) was generated from BayGenomics genetrap embryonic stem (ES) cells (see Materials and methods for a detailed description). *Trim37* mRNA expression, quantified by qRT-PCR in the mixed SV129/C57BL *Trim37^−/−^* strain, was reduced to 0.7–1.0% in brain (*P*=0.00014) and to 0.5–3.1% in testis (*P*=0.000015) of *Trim37^−/−^* compared to wild-type mice (Fig. S1C). A congenic *Trim37^−/−^* mouse strain in C57BL/6JOlaHsd with wild-type mice of the same background strain as controls were used in all subsequent experiments.

**Fig. 1.**
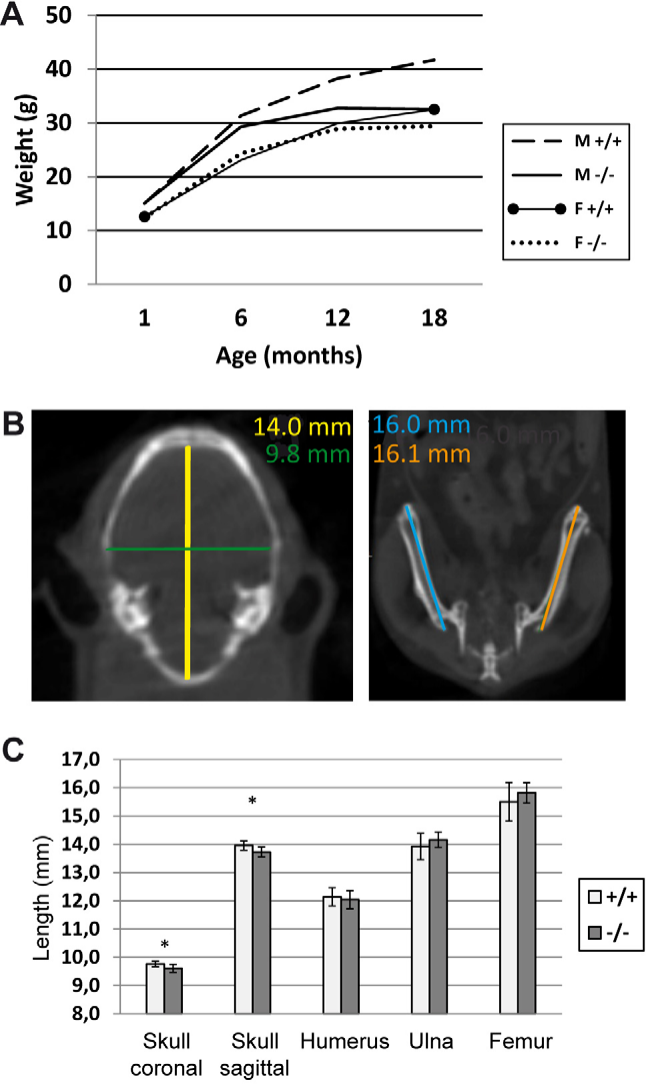
**Weight and bone parameters in *Trim37*^−/−^ mice.** (A) Weight development in *Trim37^−/−^* and control mice, age 1 to 18 months; mean±s.d. and group size (*n*) at given age points 1, 6, 12 and 18 months. M+/+: 15.0±2.9, *n*=17; 31.4±3.7, *n*=16; 38.3±5.0, *n*=4; 41.7±6.3, *n*=4; M−/−: 15.1±2.5, *n*=12; 29.3±2.8, *n*=15; 32.8±7.9, *n*=6; 32.5±5.3, *n*=4; F+/+: 12.6±1.5, *n*=21; 23.1±2.2, *n*=14; 29.9±4.1, *n*=8; 32.5±4.8, *n*=8; F−/−: 12.3±1.6, *n*=21; 24.3±2.4, *n*=15; 28.9±3.3, *n*=8; 29.4±2.6, *n*=7. Early weight development of *Trim37^−/−^* mice is normal in both males and females. The males start losing weight at the age of 6 months and the females at the age of 12 months. (B) CT scan images of skull and femurs showing the measurement of sagittal (yellow line) and coronal (green line) skull width and femur length (blue and orange lines). (C) Skull size of six-month-old *Trim37^−/−^* mice is significantly smaller compared to control mice: sagittal width 9.6±0.1 mm vs 9.8±0.1 ng/ml and coronal width 13.7±0.2 mm vs 14.0±0.2 mm (mean±s.d.; *P*=0.01; *t*-test; *n*=10/genotype). No difference in the lengths of humeri, ulnae and femurs could be detected between *Trim37^−/−^* and control mice. M, male; F, female.

*Trim37^−/−^* mice were viable with no visible abnormalities (fur, whiskers, eyes, general appearance and behavior normal). Homozygous *Trim37^−/−^* mice were infertile (see below), but in heterozygous matings, pups were born with normal Mendelian frequency (Table S1). Early weight development in both sexes was normal. Male mice started losing weight at the age of 6 months and females after the age of 12 months (Fig. 1A). In computer tomography (CT) analysis, six-month-old *Trim37^−/−^* mice had significantly smaller than normal skull size, but no difference in the lengths of long bones was seen (Fig. 1B,C).

After the age of 12 months approximately one third of mice started to show increasing problems in well-being, appearing as weight loss of up to 20%, reduced mobility and changes in behavior. Gross pathologic examination of these animals revealed hepatomegaly and fatty liver, splenomegaly, enlargement of pancreas with a granular appearance, ascites and tumors. The most severe problems appeared in animals over 18 months of age (see Table S2 for details), which was set as the latest time point for euthanasia. Extensive behavioral phenotyping did not show any major differences between *Trim37^−/−^* and control mice (data not shown).

### *Trim37^−/−^* mice are infertile due to gonadal degeneration

At visual examination both male and female *Trim37^−/−^* mice had normal external genitalia. The mice were mating in a normal manner as visualized by female plugs after mating, indicating normal masculinization of *Trim37^−/−^* males. Male *Trim37^−/−^* mice did not reproduce at any age (post-natal week 6 onwards). Female *Trim37^−/−^* mice were able to become pregnant at the age of 6 weeks, but females older than that were infertile.

**Fig. 2.**
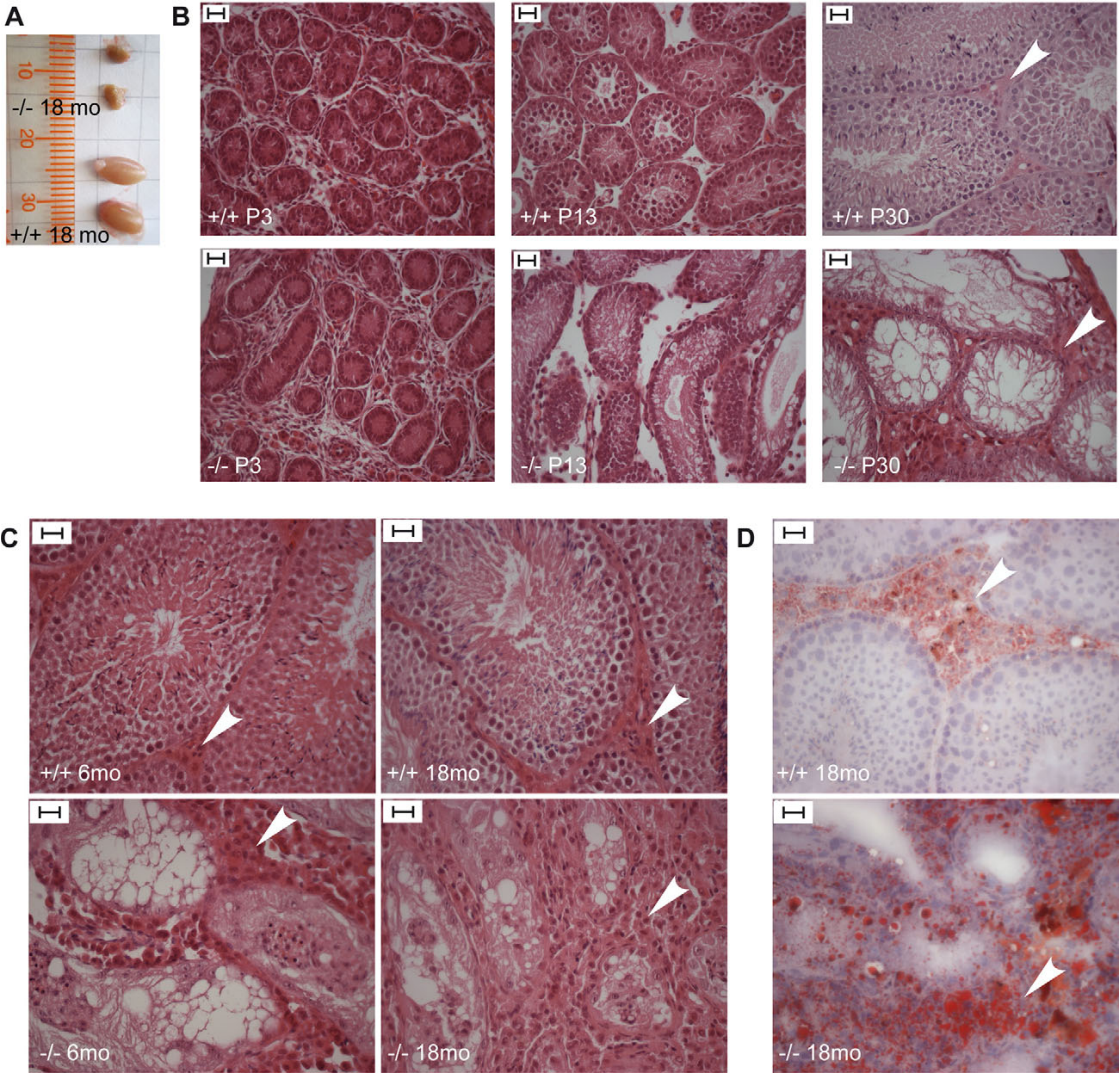
**Testicular findings in *Trim37^−/−^* males.** (A) Testicles of a 1.5-year-old *Trim37^−/−^* mouse and a wild-type littermate. The size of *Trim37^−/−^* testicles is reduced to approximately 30% of that of wild-type mice. (B) HE-stained sections demonstrating degeneration of germ cells and Leydig cell hyperplasia in *Trim37^−/−^* mouse testis, starting at P13. Total germ cell aplasia is evident in one-month-old mice. (C) Adult *Trim37^−/−^* mice show total germ cell aplasia and Leydig cell hyperplasia that is increasing with age. Leydig cells are marked with white arrowheads. (D) ORO stained testicular sections from 1.5-year-old mice, showing excessive intracellular and intercellular lipid accumulation around the area of Leydig cells in the *Trim37^−/−^* mice testis. Scale bars: 20 µm.

*Trim37^−/−^* males showed histological evidence of testicular degeneration already at postnatal day 13 (P13) and in adult mice older than 6 months the testis size was approximately 30% of that in control mice (Fig. 2A). On histological level the testes of *Trim37^−/−^* mice showed degeneration of germ cells and Leydig cell hyperplasia starting at P13 with total absence of germ cells being evident in one-month-old mice (Fig. 2B). Leydig cell hyperplasia in *Trim37^−/−^* mice increased with age (Fig. 2B,C). The testes showed progressive neutral fat accumulation evaluated as Oil Red O (ORO) staining in and around Leydig cells from one month onwards (data not shown), with excessive lipid accumulation present at 18 months (Fig. 2D). Hormonal measurements demonstrated that four-month-old male *Trim37^−/−^* mice had elevated levels of follicle-stimulating hormone (FSH; 83.0±12.8 ng/ml in *Trim37^−/−^* vs 41.7±17.4 ng/ml in controls; *P*=0.0014) and elevated levels of luteinizing hormone (LH; 8.53±2.8 ng/ml in *Trim37^−/−^* vs 1.85±2.19 ng/ml in controls; *P*=0.007; *n*=4–6/genotype in both measurements). No difference was seen in levels of testosterone between *Trim37^−/−^* mice (1.24±0.45 nmol/l) and controls (1.13±0.31 nmol/l; *n*=5/genotype).

**Fig. 3.**
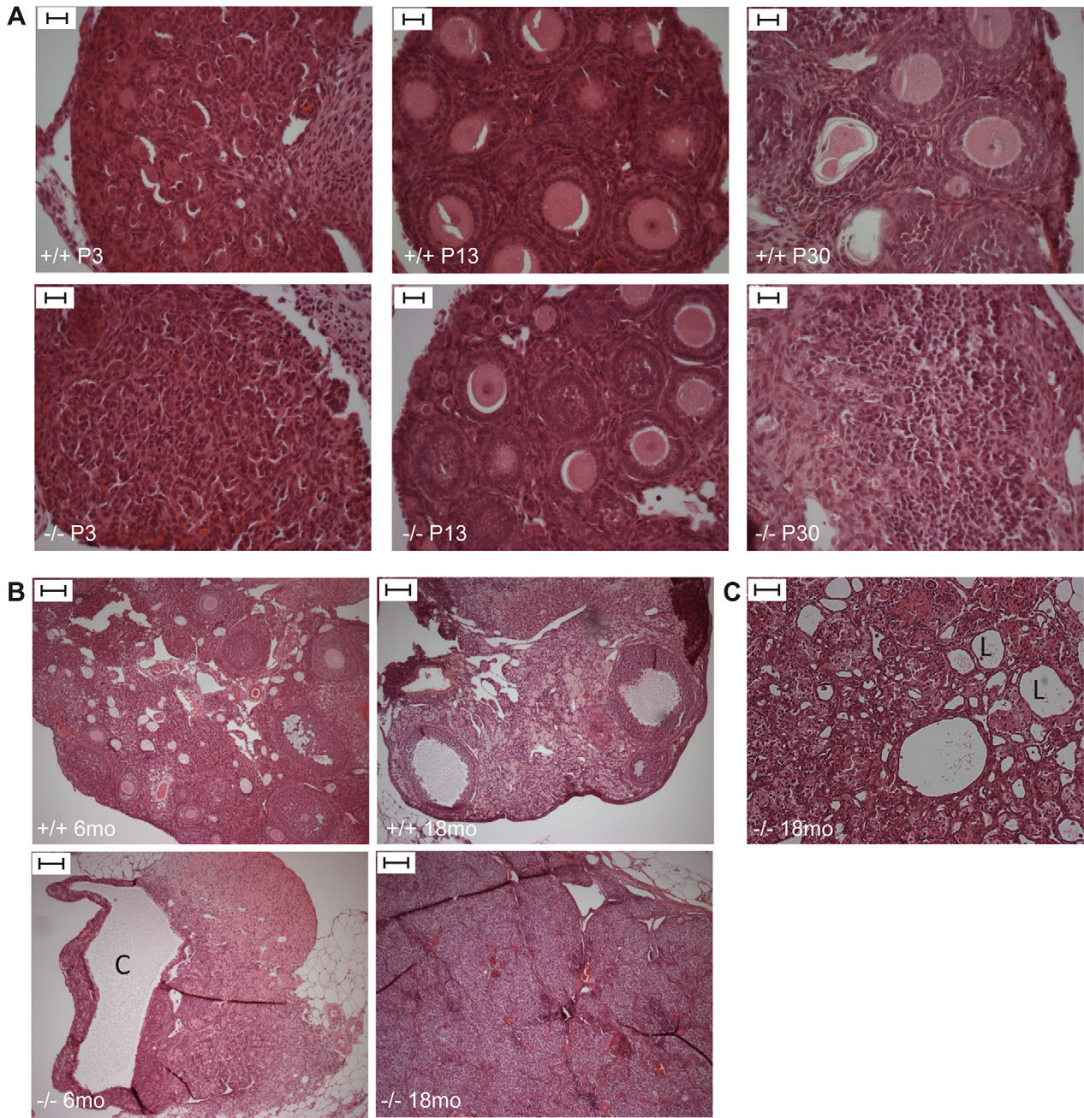
**Ovarian histology in *Trim37^−/−^* females.** (A) HE-stained sections demonstrating reduced number of oocytes in ovaries of *Trim37^−/−^* mice at P30. Scale bars: 20 µm. (B) HE-stained sections of ovaries in adult mice showing germ cell aplasia and hilus cell hyperplasia in the *Trim37^−/−^* mouse. One cyst (C) is visible in the 6-month-old *Trim37^−/−^* female. Scale bar 100 µm. (C) Some *Trim37^−/−^* mice presented with ovarian lipid cell (L) hyperplasia. Scale bars: 20 µm.

Histological analysis of *Trim37^−/−^* ovaries demonstrated germ cell aplasia and hilus (morphologically similar to Leydig cells of the testes) cell hyperplasia (Fig. 3A,B). In the developing ovary primordial follicles and oocytes were present, but already in one-month-old *Trim37^−/−^* mice the number of oocytes was low, when compared to wild-type controls (Fig. 3A). At the age of six months female *Trim37^−/−^* mice showed absence of germ cells. Some females also showed ovarian lipid cell hyperplasia (Fig. 3C). In addition, common ovarian findings in *Trim37^−/−^* mice included cysts and Sertoli cell tumors (Fig. 3B, for more details, see paragraph ‘Increased incidence of ovarian Sertoli cell tumors in *Trim37^−/−^* females’).

### *Trim37^−/−^* mice show non-compaction cardiomyopathy

**Fig. 4.**
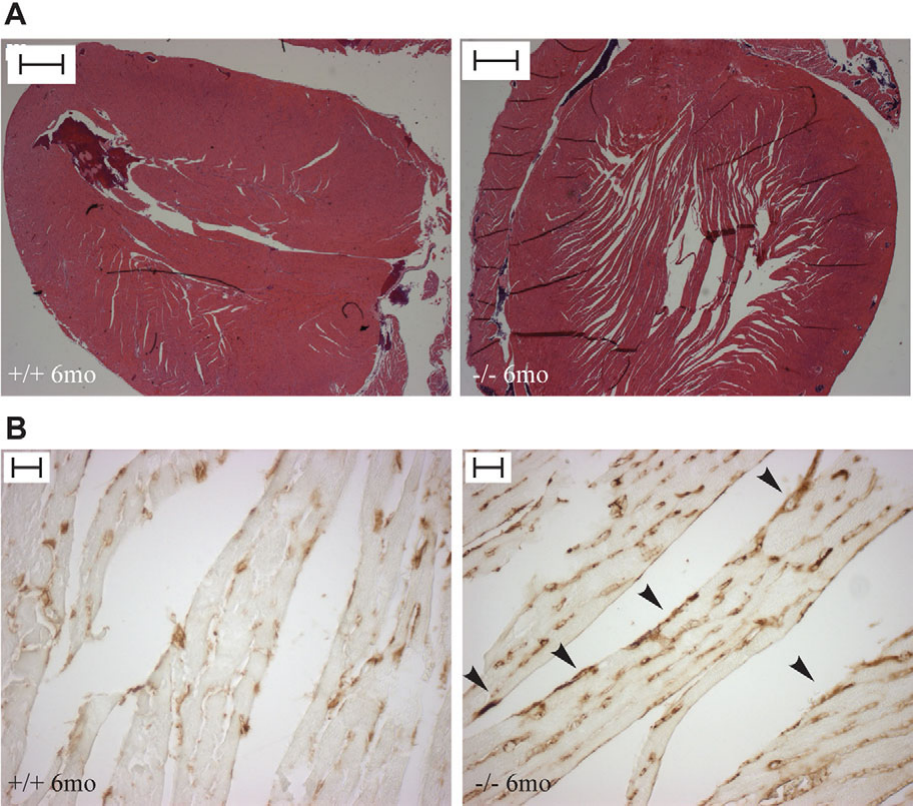
**Non-compaction cardiomyopathy in *Trim37^−/−^* mice.** (A) HE-staining of heart sections show hypertrabeculation of the heart in six-month-old *Trim37^−/−^* mice. Scale bars: 500 µm. (B) Endothelial staining (anti-CD34 immunostaining) is present in the trabeculations of the ventricular wall in *Trim37^−/−^* mice indicative of non-compaction cardiomyopathy (arrowheads). Scale bars: 20 µm.

Histological analysis of hematoxylin-eosin (HE)-stained sections of heart revealed myocardial hypertrabeculation indicative of non-compaction cardiomyopathy (Fig. 4A). The majority of *Trim37^−/−^* mice had more trabeculated ventricular walls compared to wild-type controls. Immunohistochemical staining with anti-CD34 showed the presence of endothelial cells in the trabeculations of the ventricular wall (Fig. 4B). The weight of heart/total body weight of *Trim37^−/−^* mice did not differ from wild-type controls (data not shown).

### Fatty liver disease and disturbed glucose metabolism in *Trim37^−/−^* mice

**Fig. 5.**
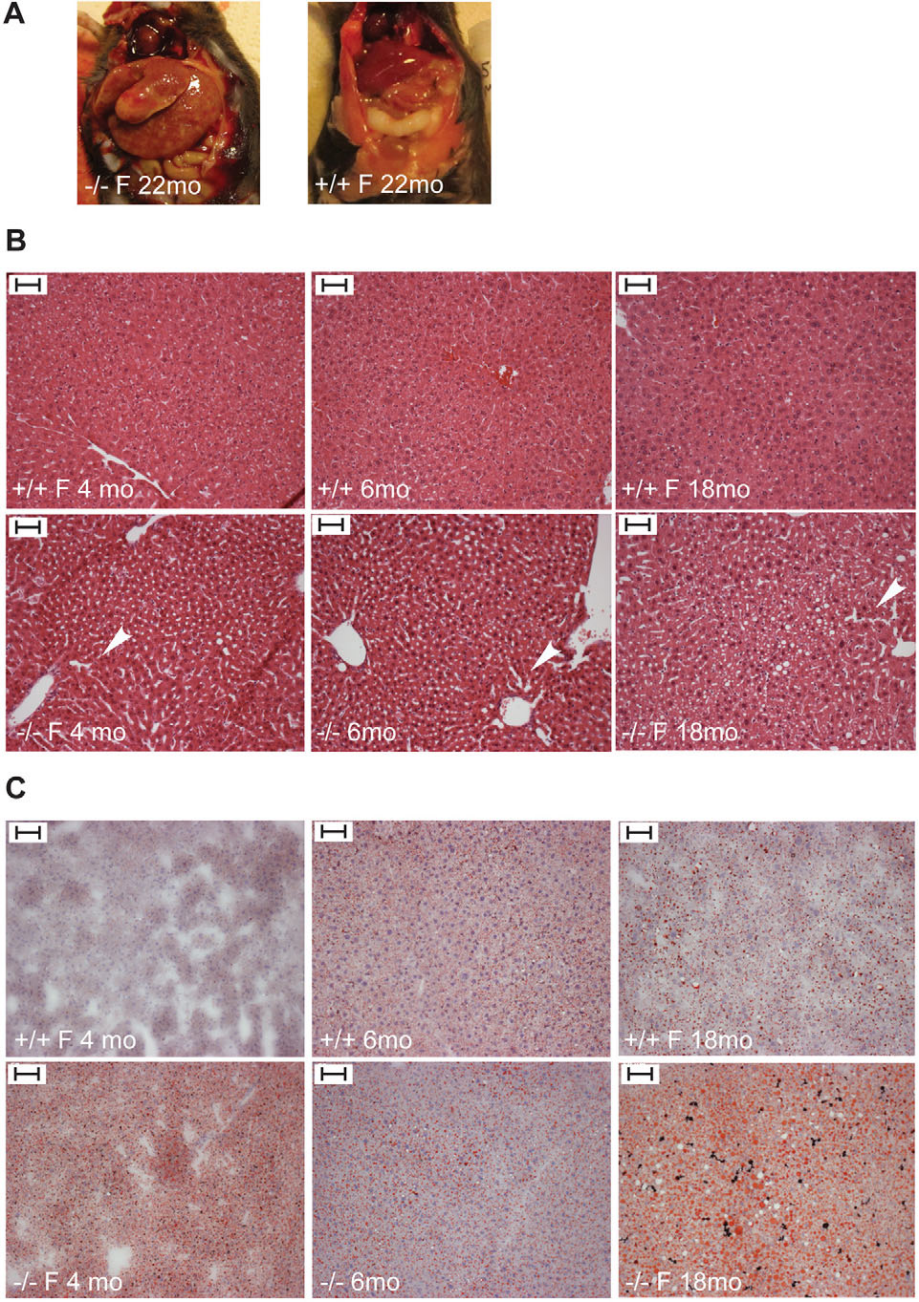
**Hepatomegaly and fatty liver in *Trim37^−/−^* mice.** (A) Enlarged liver and difference in the liver color in a 22-month-old *Trim37^−/−^* female (F) mouse observed in gross pathological examination. (B) HE-staining of liver sections demonstrating sinusoidal dilatation (white arrowheads) and vacuolization of *Trim37^−/−^* liver. (C) Accumulation of neutral lipids in *Trim37^−/−^* liver visualized by ORO staining. Scale bars: 50 µm.

Aged *Trim37^−/−^* mice (22–24 months) showed a tendency towards enlarged relative liver size with prominent hepatomegaly (liver weight 3.0 g and 5.6 g, compared to a mean of 1.5 g in wild-type mice) in a subset of female mice (Fig. 5A; Fig. S2A). Histological analysis of HE-stained liver sections in 1-, 4-, 6- and 18-month-old mice showed progressive vacuolization of the liver (Fig. 5B). One-month-old *Trim37^−/−^* mice and wild-type littermates had similar liver histology, but at the later age points *Trim37^−/−^* mice liver sections contained increasing amounts of vesicles. The vesicles were confirmed to have neutral lipid (majority triacylglycerols) accumulation in droplets by staining with ORO in liver cryosections (Fig. 5C). The ORO staining data was consistent with the results from HE-staining with *Trim37^−/−^* mice older than 1 month showing neutral lipid accumulation in liver, with the staining intensity and lipid droplet size increasing with age. The lipid accumulation was more prominent in females than in males. Quantitative analysis of triacylglycerols extracted from liver tissue (*n*=4–8/group) (Fig. S2B,C) showed that lipid accumulation in liver started approximately at the age of 4–6 months and increased with age, being more prominent in females than in males. The variation in the content of liver triacylglycerols was high between animals, even within the same genotype/gender group (Fig. S2B,C).

Analysis of ORO stained cryosections from brain, pancreas, adrenal gland and ovary did not show any difference in staining pattern or intensity between *Trim37^−/−^* mice and controls (data not shown). White adipose tissue cell size, determined from images captured from HE-stained paraffin embedded sections, increased with age, but no difference in cell size was seen between *Trim37^−/−^* mice and wild-type controls (data not shown).

We next assessed the lipid status and several other clinical parameters in serum samples of six-month-old *Trim37^−/−^* and control male mice after 24-h fasting. Adult six-month-old *Trim37^−/−^* mice had elevated fasting blood glucose levels (4.71±0.73 mmol/l vs 3.79±0.52 mmol/l; *P*=0.005) and low fasting serum insulin values (0.37±0.12 ng/ml vs 0.59±0.33 ng/ml; *P*=0.066) compared to wild-type littermates (Table 1). Histological analysis of HE-stained pancreatic tissue sections demonstrated similar amount, size and distribution of pancreatic β-islets in *Trim37^−/−^* mice and controls. No difference was detected in immunohistochemical staining with an anti-insulin antibody in pancreas (data not shown). No difference between *Trim37^−/−^* and control mice in serum triacylglycerol, cholesterol, free fatty acids (FFA), long chain fatty acids, very long chain fatty acids, branched chain fatty acids or the other assesses parameters were observed (Table 1).

**Table 1.**
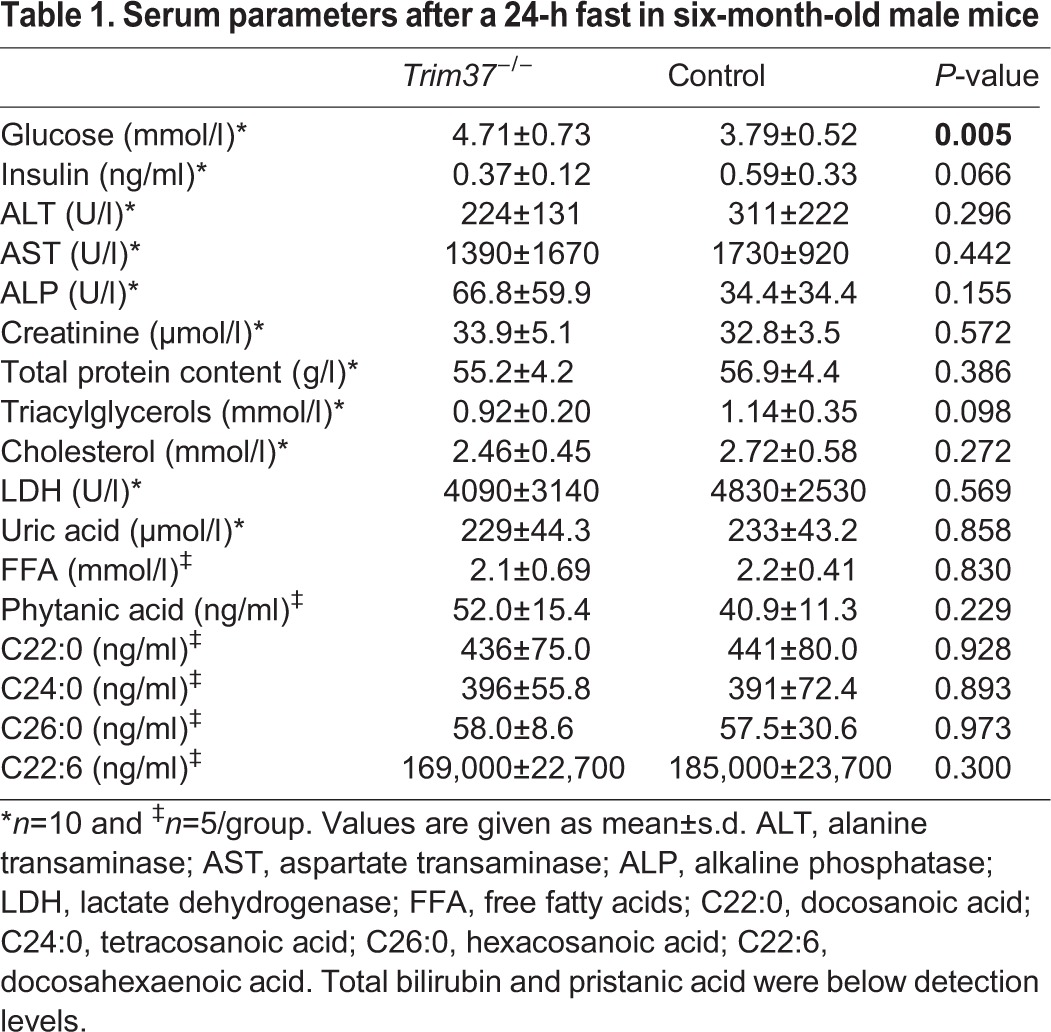
**Serum parameters after a 24-h fast in six-month-old male mice**

Metabolic phenotyping using the Comprehensive Laboratory Animal Monitoring System (CLAMS; see Materials and methods for details) was performed for 30 mice (*n*=3–4/group; age 20 weeks), fed either standard or fat-enriched Western diet since weaning. There was no effect of genotype or interaction between genotype and diet on the measured parameters (Table S3). The only differences observed were related to the diet, with, as expected, the mice on high-fat Western diet being heavier than control mice. However, the mice on Western diet also showed reduced food intake (males), reduced water consumption, reduced respiratory exchange ratio (RER), and lower physical activity (Table S3).

### Increased incidence of ovarian Sertoli cell tumors in *Trim37^−/−^* females

**Fig. 6.**
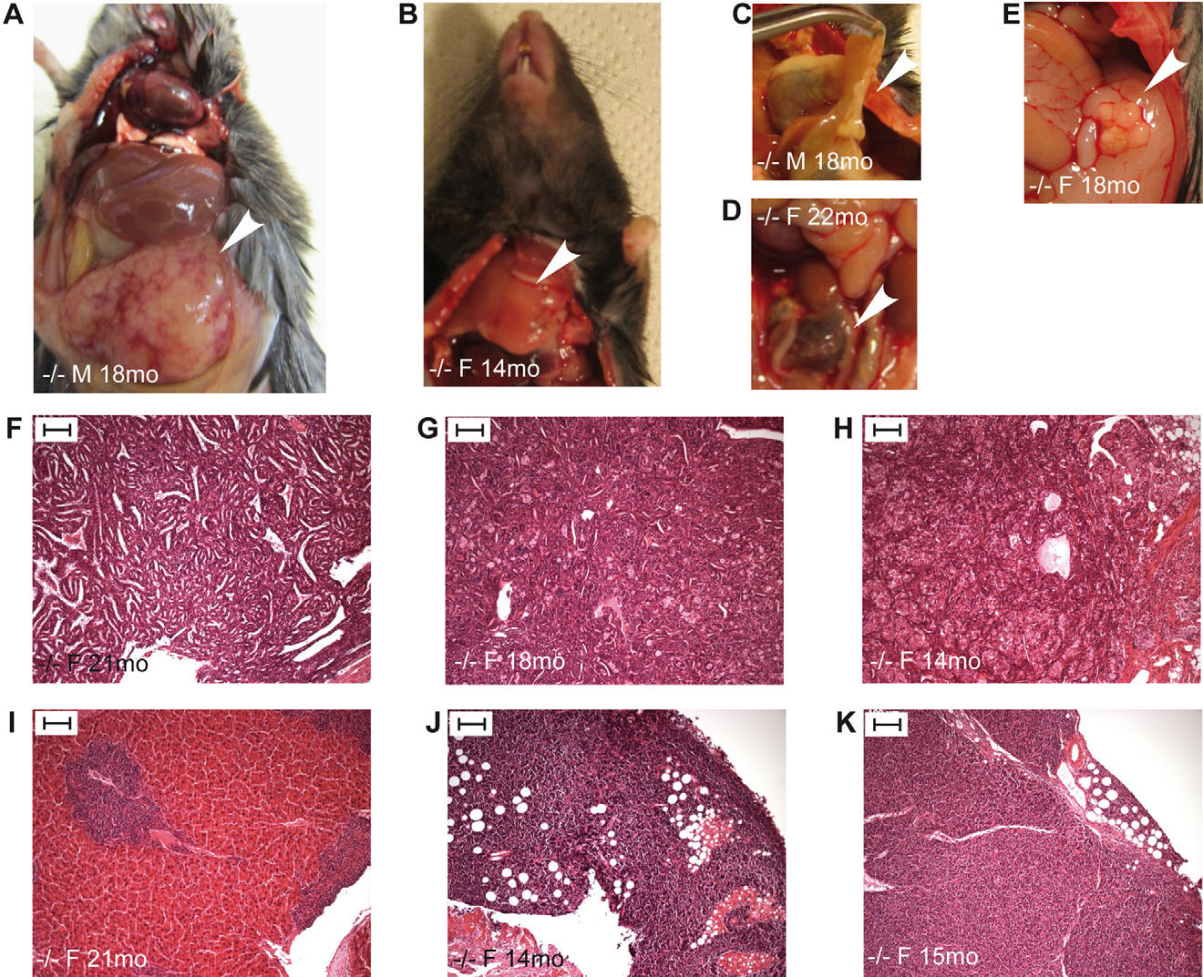
**Examples of macroscopic findings in pathological gross examination and tumor histology in *Trim37^−/−^* mice.** (A) Enlargement of spleen (arrowhead) in an 18-month-old male (M) mouse. The same mouse had lymphoma of the lymph nodes. (B) Enlarged thymus (arrowhead) in a 14-month-old female (F) mouse. The tumor was confirmed to be a neuroendocrine carcinoma in histological examination (J). (C) Enlarged pancreas (arrowhead) showing a granular appearance in an 18-month-old male mouse. In histological examination the pancreatic duct was shown to be cystic, but no tumor formation was present. (D) Abnormal tissue around the ovary (arrowhead) in a 22-month-old female mouse. In histological examination, the ovary was shown to be hemorrhagic and necrotic, possibly presenting ovarian torsion. (E) Ovarian tumor (arrowhead) in an 18-month-old female mouse. The tumor was in histological examination confirmed to be of Sertoli cell type (G). (F–K) HE-staining of tumor sections. Ovarian retiformic Sertoli-cell tumor, female 21 months (F). Ovarian Sertoli cell tumor, female 18 months (G). Ovarian Sertoli cell tumor, female 14 months (H). Primitive neuroendocrine liver tumor, female 21 months (I). Neuroendocrine carcinoma of the thymus, female 14 months (J). Lymphoma found in the abdomen, female 15 months (K). Scale bars: 100 µm. M, male; F, female.

A gross pathological examination was performed for all *Trim37^−/−^* mice (*n*=204) and wild-type controls (*n*=192), 1 to 18 months of age, dissected during this study (Fig. 6A–E). Histological samples were collected from all tumors and tissues with abnormal appearance. Tumors were detected in eighteen *Trim37^−/−^* mice and two wild-type controls (Table 2). With the exception of one tumor, all were detected in over one-year-old animals and the majority of them were ovarian tumors of Sertoli cell type (Table 2, Fig. 6F–H). In addition, there were four individual *Trim37^−/−^* mice with other tumors (Table 2, Fig. 6I–K). Two of the *Trim37^−/−^* mice presented with multiple tumors of the same type in different locations of the body.

**Table 2.**
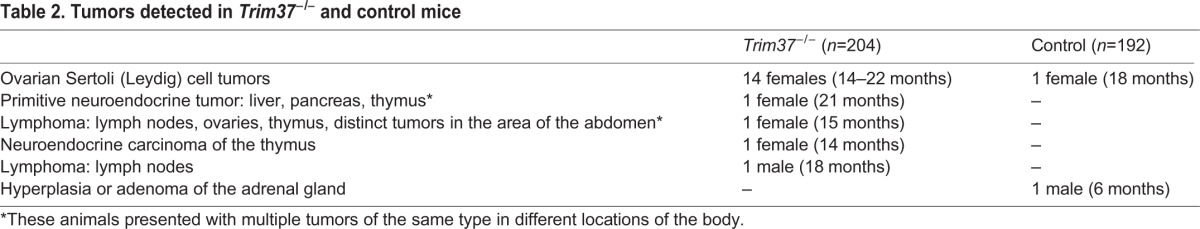
**Tumors detected in *Trim37^−/−^* and control mice**

### *Trim37^−/−^* mice do not display overt peroxisomal pathology

Liver tissue of three-month-old female *Trim37^−/−^* mice and wild-type controls (*n*=3/genotype) was examined by electron microscopy (EM) with focus on morphology and distribution of peroxisomes. Observation of mouse liver electron micrographs gives a cross estimation over peroxisome abundance. No difference was seen in the size and shape of peroxisomes between the genotypes, but *Trim37^−/−^* liver contained more lipid droplets that were larger in size, when compared to control tissue (Fig. S3A).

The response of peroxisomes to a well-known peroxisomal proliferator, clofibrate, was assessed in groups of four-month-old male mice fed with a diet containing 0.5% (w/w) clofibrate for two weeks. Clofibrate is a well-documented lipid lowering drug, which increases lipoprotein lipase activity and induces peroxisomal proliferation, by increasing the amount and size of peroxisomes in the liver of rodents (Lazarow and De Duve, 1976). Peroxisomal multifunctional enzyme type 1 (MFE-1) is a protein that is highly inducible by hypolipidemic drugs, which occurs in parallel with proliferation of peroxisomes. The effect of clofibrate was assessed by immunoblotting liver lysates with anti-MFE-1 antibody, (Fig. S3B). The amounts of MFE-1 in the control group (normal diet) and in mice euthanized immediately after the two-week feeding period with clofibrate (Cf+0) were similar in both genotypes, suggestive of similar peroxisomal proliferation (Fig. S3B). No difference was seen in the clearance of MFE-1 after the treatment, as the amount of MFE-1 was similar in *Trim37^−/−^* and control mice five days after cessation of the clofibrate diet (Cf+5; Fig. S3B).

Extraction of lipids from livers of the clofibrate treated mice showed a tendency for *Trim37^−/−^* mice to respond in a more efficient manner to increased lipoprotein lipase activity due to clofibrate treatment, although no statistical significance was reached (liver triacylglycerols in Cf+0 groups: *Trim37^−/−^* 19.6±6.3 vs wild-type mice 34.4±5.5; *P*=0.065) (Fig. S3C). This finding was further supported by immunoblotting liver lysates from clofibrate-treated and control mice with an antibody recognizing adipose differentiation-related protein (ADRP) (Fig. S3D). ADRP is a lipid droplet-associated protein, expressed early during adipocyte differentiation and is a specific marker of lipid accumulation (Imamura et al., 2002). Quantification of the ADRP intensities demonstrated that there was a tendency to an increased lipid lowering response to clofibrate in *Trim37^−/−^* compared to control mice (Fig. S3D).

Finally, in order to investigate if there is a difference in peroxisomal β-oxidation efficiency we measured the levels of long chain fatty acids, very long chain fatty acids and branched chain fatty acids in the sera of the clofibrate treated mice (Table S4). No differences could be detected between *Trim37^−/−^* and wild-type mice in levels of phytanic acid, docosanoic acid, tetracosanoic acid, hexacosanoic acid or docosahexaenoic acid. Pristanic acid values were below detection levels, except in five *Trim37^−/−^* mice (1.5–7.5 ng/ml). Thus, pristanic acid was below the detection level in all wild-type mice (Fig. S3E).

## DISCUSSION

The *Trim37*-deficient mouse model recapitulates several key features of the multi-organ human disorder Mulibrey nanism, including infertility, cardiomyopathy, fatty liver and risk of various tumors (see Table S5 for the approximate frequency of clinical manifestations in human patients and *Trim37^−/−^* mice). Constant findings that were seen in all *Trim37^−/−^* mice were infertility and a small skull size. The other features were more variable between mice. Also in MUL patients, organ manifestations differ, especially in childhood (Karlberg et al., 2004a). Not all patients develop all symptoms and the severity of the disease varies even between Finnish patients homozygous for the same mutation (Karlberg et al., 2004a).

The most consistent phenotypes in human MUL patients are growth restriction and characteristic craniofacial features (Karlberg et al., 2004a). The growth restriction is prenatal-onset and there is incomplete catch-up growth with a final adult height on average of 136 cm in females and 150 cm in males (Karlberg et al., 2007). The craniofacial features are characterized by a large skull, triangular face, broad forehead, low nasal bridge and scaphocephaly with occipital bossing (Karlberg et al., 2004a). In contrast, *Trim37^−/−^* mice showed a decrease in skull size, whereas no changes in the length of the long bones were detected. This is, however, not unexpected given that transgenic mice seldom recapitulate growth restriction phenotypes encountered in human patients. For example missense mutations in genes involved in fibroblast growth factor (FGF) signaling in humans lead to various congenital growth restriction phenotypes, whereas mouse models for these disorders show normal skeletal phenotypes (Su et al., 2014). Neither could we in *Trim37^−/−^* mice identify structural abnormalities such as fibrous dysplasia of the long bones, encountered in approximately one fourth of the human patients (Karlberg et al., 2004a).

Infertility is one of the central phenotypes in human MUL (Karlberg et al., 2004b, 2011). In male patients infertility is associated with small testes size, elevated levels of FSH and LH, low inhibin B and low to normal testosterone levels (Karlberg et al., 2011). Female patients all develop premature ovarian failure and are at a high risk of developing ovarian fibrothecomas (Karlberg et al., 2004b). The infertility phenotype is recapitulated in *Trim37^−/−^* mice. Male mice were not able to reproduce at any age and females only shortly after puberty. In human MUL patients, however, no pregnancies have been conceived among the females but one male patient has fathered a child using assisted reproductive techniques (Karlberg et al., 2004b, 2011). Based on histological analysis of human and mouse gonads it is evident that TRIM37 has a critical role in regulating the maturation of both spermatogenic cells and oocytes. Both male and female *Trim37^−/−^* mice showed early germ cell degeneration with total aplasia by the age of one month in males and six months in females, as well as hilus and Leydig cell hyperplasia, which in male mice was associated with excessive intracellular and intercellular accumulation of neutral lipids. Ovaries of *Trim37^−/−^* mice also showed lipid cell hyperplasia and increased incidence of both cysts and Sertoli cell tumors. The hormonal status in adult male *Trim37^−/−^* mice is similar to the human patients (Karlberg et al., 2011), with high FSH and LH, but normal testosterone levels. The hormonal data in combination with Leydig cell hyperplasia imply the presence of compensatory feedback mechanisms to maintain sufficient Leydig cell function. The spermatogenic impairment seems therefore not to be caused by lack of testosterone but rather due to an intrinsic failure of the germ cells to survive. The increased lipid load in Leydig cells can be a sign of functional difficulties in Leydig cell steroidogenesis, which is also reflected by the elevated LH concentration.

The most severe and life-threatening manifestation of human MUL is the heart disease, manifesting as constrictive pericarditis and cardiomyopathy with hypertrophy and variable fibrosis (Lipsanen-Nyman et al., 2003). Histological analysis of mouse heart revealed cardiomyopathy also in *Trim37^−/−^* mice. The heart tissue showed hypertrabeculation that stained with the endothelial marker CD34, implying presence of non-compaction cardiomyopathy similarly to the histopathological findings seen in some of the human MUL patients (Karlberg et al., 2009a). Non-compaction of ventricular myocardium (isolated non-compaction cardiomyopathy of left ventricle) is a cardiomyopathy where the loosely interwoven fibers of embryonal myocardium fail to condense normally into solid myocardium leaving in the heart muscle deep recesses, which communicate freely with the left ventricle (Ikeda et al., 2015). Positive CD34 staining in the endomyocardium of *Trim37^−/−^* mice verified that these recesses were true anatomical structures and not artefactual splits, thus confirming the diagnosis of non-compaction. This is a significant finding, suggesting that non-compaction cardiomyopathy is an additional element in the development of the MUL heart disease. This type of non-compaction cardiomyopathy is still a relatively poorly defined entity. The consequent heart failure may be due to abnormal filling and relaxation, as both systolic and diastolic dysfunction has been described in human patients. The cardiac phenotype in *Trim37^−/−^* mice appears to be mild, as the mice were viable and survived until adulthood. A subset of mice, however, had problems in well-being. Even if autopsy in these mice showed enlargement of liver, spleen and pancreas as well as tumors, the impact of cardiopathy cannot be excluded. Echocardiography would be needed to evaluate the contribution of the cardiac pathology to cardiac function and well-being in *Trim37^−/−^* mice.

Liver manifestations are commonly seen in MUL patients. At the time of diagnosis, approximately half of the patients present with hepatomegaly and fatty liver is seen in the majority of adults and adolescents as well as in half of the prepubertal children (Karlberg et al., 2004a, 2005). In addition, almost half of the MUL patients develop hepatic peliosis, tumorous lesions comprising blood-filled hepatic sinusoids (Karlberg et al., 2009a). In *Trim37^−/−^* mice hepatomegaly was an infrequent finding by the age of 18 months and no evidence of hepatic peliosis was observed. However, progressive vacualization and neutral lipid accumulation in liver was commonly seen. Triacylglycerol accumulation in liver was shown to start at the age of 4–6 months and to increase with age, being, however, highly variable and more prominent in female than in male *Trim37^−/−^* mice. The liver findings in mice are in line with the observation that deficient TRIM37 function leads to fatty liver disease.

The most important metabolic finding in MUL is insulin resistance (Karlberg et al., 2005). While children present with both low fasting blood glucose and insulin values, more than 90% of the adult patients show abnormally high fasting insulin levels resulting in type 2 diabetes in half of the patients (Karlberg et al., 2005). *Trim37^−/−^* mice presented with low fasting serum insulin values and elevated fasting blood glucose values after a 24-h fast, implying disturbed glucose metabolism. However, the genetic background of the *Trim37^−/−^* mouse probably contributes to the low insulin values, as the C57BL/6 mouse strain carries a naturally occurring deletion in the nicotinamide nucleotide transhydrogenase gene, which results in impaired insulin secretion (Freeman et al., 2006) thus possibly overpowering the putative insulin elevating effects caused by chronic exposure of elevated glucose seen in human patients. Moreover, a 24-h fast in mice is not entirely equivalent to human overnight fasting, as it depletes all the glycogen storages in the liver and provokes a catabolic state in mice (Ayala et al., 2006). In contrast to human overnight fasting that impairs insulin stimulated glucose utilization, a prolonged fast in mice enhances it (Ayala et al., 2006; Heijboer et al., 2005). A transfer of the *Trim37^−/−^* knock-out into another mouse background strain would be needed to evaluate the glucose and insulin metabolism, including glucose tolerance tests with insulin assessment, of *Trim37^−/−^* mice without the insulin lowering effects of the nicotinamide nucleotide transhydrogenase mutation.

MUL patients are at high risk of developing both benign and malignant tumors (Karlberg et al., 2004a, 2009a). In addition, disturbed organ development and ectopic tissues are frequent findings (Karlberg et al., 2009a). The benign tumors include mainly vascular lesions, cysts, adenomatous and nodular lesions (Karlberg et al., 2009a), as well as ovarian fibrothecomas (Karlberg et al., 2009b, 2004b). The main types of malignant tumors include Wilm's tumor (Hämäläinen et al., 2006; Karlberg et al., 2004a, 2009a; Seemanova and Bartsch, 1999; Similä et al., 1980), carcinomas of different type, acute lymphoblastic leukemia and histiocytosis of Langerhans cells (Karlberg et al., 2009a). *Trim37^−/−^* mice also had an increased risk of developing tumors, showing a higher frequency of both benign and malignant tumors than wild-type control animals. Ovarian tumors of Sertoli cell type were the most frequent tumor type encountered, with other tumors such as primitive neuroendocrine tumor, lymphoma and neuroendocrine carcinoma only occasionally seen. Thus, even if there was a clear difference in the predominant tumor types between human patients and *Trim37^−/−^* mice our data further strengthens the findings in human MUL patients that deficient function of TRIM37 is associated with increased risk of tumorigenesis.

TRIM37 has been suggested to have a peroxisome-associated function due to its localization to peroxisomes (Kallijärvi et al., 2002) and due to some overlapping clinical features between MUL and peroxisomal disorders (Karlberg et al., 2004a). Peroxisomes are involved in a variety of metabolic processes such as α- and β-oxidation of long, very long, branched chain and polyunsaturated fatty acids, as well as biosynthesis of plasmalogens and bile acid (van den Bosch et al., 1992; Wanders and Waterham, 2006a). Peroxisomal disorders can be caused by deficiency of single peroxisomal enzymes (Wanders and Waterham, 2006b) or *PEX* gene defects leading to peroxisomal biogenesis disorders (PBDs) (Steinberg et al., 2006). Here we assessed the role of peroxisomes in *Trim37^−/−^* pathology using different approaches. No obvious differences in the number, structure or distribution of peroxisomes in liver tissue between *Trim37^−/−^* and control mice were identified. Moreover, both the proliferation and clearance of the peroxisomal marker MFE-1 seemed to be similar between *Trim37^−/−^* and control mice upon treatment with clofibrate, a lipid-lowering agent that increases lipoprotein lipase activity and induces peroxisomal proliferation. Interestingly, analysis of triacylglycerol content and ADRP protein amount suggest that *Trim37^−/−^* mice may respond to clofibrate by more efficient lipase activity as in *Trim37^−/−^* mice liver both triacylglycerol and ADRP levels were lower compared to the wild-type controls immediately after clofibrate treatment. Finally, no difference was seen in levels of long, very long and branched chain fatty acids between *Trim37^−/−^* and control mice, with the exception of pristanic acid, which was measurable in a subset of *Trim37^−/−^* mice, the values being below detection level in all wild-type controls. This could reflect a difference in peroxisomal β-oxidation efficiency between *Trim37^−/−^* and wild-type mice. Peroxisomal β-oxidation has previously been shown to be essential for lipid homeostasis and male fertility (Baes and Van Veldhoven, 2012; Huyghe et al., 2006) and would thus be in line with the identified testicular degeneration and lipid accumulation in *Trim37^−/−^* mice. Of note, deficiencies in peroxisomal function can result in novel and unexpected phenotypes. For example, genetic variation in the transcriptional regulation of peroxisomal and mitochondrial fatty acid oxidation has been reported to contribute to the development of left ventricular hypertrophy (Jamshidi et al., 2002). Moreover, the absence of *Pxmp2* that is a channel-forming protein in peroxisomal membrane leads in failure of mammary gland development in puberty (Vapola et al., 2014).

In summary, the *Trim37^−/−^* mouse provides a good model to study the pathogenetic mechanisms associated with TRIM37 deficiency. In addition to studies of MUL pathogenesis, this mouse model can prove insights into the development of infertility, non-alcoholic fatty liver disease, cardiomyopathy and tumorigenesis. These should be explored in the light of the recently implied roles of TRIM37 in centriole biogenesis and histone H2A-associated oncogenesis (Balestra et al., 2013; Bhatnagar et al., 2014).

## MATERIALS AND METHODS

### Generation of the *Trim37^Gt(bgeo)FH^* mouse strain and genotyping

A BayGenomics genetrap ES cell line with the *Trim37* gene trapped (*Trim37^Gt(RRO307)Byg^*; RRO307) was purchased from Mutant Mouse Regional Resource Centers supported by NIH, MMRRC (UC Davis, CA). The >16 kb β-geo insertion vector was shown to localize in intron 2 of *Trim37* (Fig. S1A) by polymerase chain reaction (PCR). Chimeric mice were produced by microinjection of the ES cells to C57BL/6 blastocysts, which were transferred into SV129 recipient females (Biocenter Oulu, Transgenic core facility, University of Oulu, Finland). The mixed SV129/C57BL strain was backcrossed repeatedly (>N9 generations) to C57BL/6JOlaHsd and congenity of the *Trim37^Gt(bgeo)FH^* strain (*Trim37*^−/−^) was verified by SNP genotyping using Mouse Medium Density Linkage Panel (Illumina, San Diego, CA).

Genotyping of mice was done with one forward primer: IVS2-F7 (5′-ATCCGTCTGCTGCTTTCCAT-3′) and two reverse primers: IVS2-R7 (5′-AATGCCACCTGCCTATGTCC-3′) and GT5-R7 (5′-GTTCATATGGTGCCGTGCAG-3′) (Fig. S1A-B), which enables simultaneous detection of the 350 bp wild-type and the 255 bp knock-out alleles. Genotyping of mice was performed from ear or tail samples by use of standard PCR methods.

### Quantitative reverse transcriptase analysis of *Trim37^−/−^* and control mice

*Trim37* expression in the mixed SV129/C57BL strain was quantified by TaqMan reverse transcriptase (RT) PCR (ABI PRISM 7700 Sequence Detection System, Life Technologies, Carlsbad, CA). RNA was extracted from brain and testis (*n*=3/genotype) and converted into cDNA by use of the High Capacity cDNA Archive kit (Applied Biosystems, Foster City, CA). Amplification was performed with primers 5′-TCAGGAGGTAGAACATCAG-3′ and 5′-GGACAGGTGTGGTGACAAAGGATGC-3′. Probe 6-FAM-CAGCTCGCTCTTACTGCAAGA-MGB was used for the TaqMan detection. Normalization of gene expression was performed against the endogenous control TBP (TATA-box binding protein, Applied Biosystems).

### Animal experiments

All animal experiments were approved by the National Animal Experiment Board in Finland (licenses ESLH-2006-10696/Ym-23, ESLH-2009-09811/Ym-23 and ESAVI/6691/04) and performed in accordance with good practice in laboratory animal handling, following the principles of 3Rs (replacement, reduction, refinement). The *Trim37^Gt(bgeo)FH^* strain was housed in the Laboratory Animal Center of the University of Helsinki (license ESLH-2007-05823/Ym-23). The mice were kept in controlled room temperature and had a 12:12-h light cycle, with free access to standard chow (Teklad 2916, Harlan Laboratories, Indianapolis, IN) and water. The mice were inspected daily for changes in behavior or other signs of pain, distress or disease. Criteria for euthanizing animals were weight loss of 20%, inability to obtain feed or water, abnormal behavior or movement, signs of tumors, infection or other disease symptoms. *Trim37^−/−^* mice were monitored for fertility and body weight was measured on a regular basis. In all experiments gender matched wild-type littermates were used as controls.

### Skeletal assessment with computer tomography

Computer tomography (CT) scans were performed at the Viikki *in vivo* imaging unit (http://www.helsinki.fi/cdr/core/nanospect-ct.html), to assess skeletal parameters in six-month-old mice (*n*=10/genotype). The CT scan images were captured with the NanoSPECT-CT device (Bioscan Inc., Washington, DC) in mice anesthetized with isoflurane (4% induction, 2% maintenance). The images were used for measurement of sagittal and coronal skull size and length of femur, humerus and ulna.

### Histology

Brain, heart, lung, liver, pancreas, spleen, intestine, kidney, adrenal gland, ovary, testis, rib, femur and white adipose tissue, as well as tumors, were collected from mice at the age of 1, 4, 6 and 18 months. Tissues for one panel, obtained from mice euthanized by cervical dislocation, were snap frozen in liquid nitrogen after dissection and stored in −80°C. Tissues for a second panel were perfusion fixed in mice under terminal anesthesia with pentobarbital (Mebunat Vet 60 mg/ml, Orion, Finland), followed by cardiac perfusion with 4% paraformaldehyde (PFA). After dissection the tissues were post-fixed in 4% PFA and embedded in paraffin. Samples of rib and femur were decalcified before embedding in 0.5 M ethylenediaminetetraacetic acid (EDTA), pH 7.8 for one and two weeks, respectively. Paraffin sections of 4 µm were cut and stored in +4°C prior to staining.

For basic histological analysis of tissues standard HE staining was performed. Paraffin-embedded sections of heart were stained with the endothelial marker anti-CD34 (EP373Y, ab81289; Abcam, Cambridge, UK). The sections were pretreated with Target Retrieval Solution (DAKO, Glostrup, Denmark) and stained with Novolink Polymer Detection System (Leica Biosystems, Wetzlar, Germany), using a 1:300 antibody dilution. Insulin staining of paraffin-embedded sections of pancreas was performed with 1:800 diluted insulin (C27C9) rabbit mAb (Cell Signaling Technology, Danvers, MA). Heat-induced antigen retrieval was performed in 10 mM citrate buffer (pH 6.0). Immunostaining was performed by use of Vectastain ABC Elite kit (Vector Labs, Burlingame, CA) and SIGMAFAST 3,3′-diaminobenzidine (Sigma-Aldrich, St. Louis, MO).

For ORO staining 8–10 µm frozen sections of testis, ovary, liver, pancreas, adrenal gland and brain from mice at the age of 1, 4, 6 and 18 months were cut with the Leica CM3050 S cryotome (Leica Microsystems, Wetzlar, Germany). Chamber temperature was adjusted according to the tissue cut; temperatures between −18°C to −20°C were used. The slides with frozen sections were pre-incubated in 60% isopropanol and stained for 15 min in ORO (Solvent Red 27; Sigma-Aldrich; 3 mg/ml in 60% isopropanol). Excess ORO stain was removed by dipping the slides in 60% isopropanol and rinsed for 5 min with tap water. Counterstaining was done with Mayer's hemalum solution (Merck KGaA, Darmstadt, Germany). Slides were embedded with glycerol-gelatin (Sigma-Aldrich).

White adipose tissue cell size was estimated from HE-stained paraffin embedded sections. Light microscope images were captured and analyzed with Fiji image processing package (http://fiji.sc/Fiji). An area from the image was measured and divided by the number of cells within. This resulted in an estimate of cell size for each sample analyzed.

The number of pancreatic islets was estimated visually per low power field in pancreatic HE-stained sections and in sections immunostained for insulin. Also the morphology and size of the islets, as well as intensity of insulin immunostaining were assessed visually.

All histological analyses were done from 2–3 animals per analyzed group. The pathologist analyzing the histological data did this blinded to the genotype.

### Biochemistry of blood and serum samples

Tail tip and serum samples were collected from six-month-old male mice after 24-h fasting (*n*=10/genotype). The animals in the 24-h fasting group were housed alone and had reversed light-dark cycle for a two-week period prior to sampling. The mice were euthanized with cervical dislocation and terminal blood samples were drawn and allowed to coagulate for at least 20 min at room temperature after which serum was separated by centrifugation (10 min, 2000× ***g***).

Glucose was measured from the tail tip with the Freestyle lite freedom blood glucose monitor (Abbott Diabetes Care Inc., Alameda, CA). Insulin values were determined from the serum samples with the Ultra Sensitive Mouse Insulin ELISA kit (Chrystal Chem Inc., Downers Grove, IL). Clinical chemistry analyses, i.e. measurements of alanine transaminase (ALT), alkaline phosphatase (ALP), aspartate transaminase (AST), total bilirubin, creatinine, lactate dehydrogenase (LDH), total protein content, triacylglycerols, cholesterol and uric acid, were carried out at the Clinical Chemistry Core Facility at the Institute of Biomedicine, University of Helsinki using the ADVIA 1650 Chemistry System (Siemens AG Healthcare, Erlangen, Germany).

Non-esterified FFAs were determined by use of WAKO NEFA-HR(2) enzymatic colorimetric assay (Waco Chemicals GmbH, Neuss, Germany). Long chain fatty acids, very long chain fatty acids and branched chain fatty acids were determined as fatty acid methyl esters (FAME) by gas chromatography mass spectrometry (GC-MS) at Metabolomics, VTT Technical Research Centre of Finland. Serum samples (20 µl) were diluted with 20 µl of 0.9% sodium chloride, spiked with internal standards (TG(17:0/17:0/17:0); FFA 17:0) and extracted with chloroform:methanol (2:1 v/v; 400 µl) containing 0.1% BHT (butylated hydroxytoluene) by vortexing for 5 min. After 30 min standing, the samples were centrifuged at 10620×***g*** for 5 min and the lower layer was separated and evaporated into dryness under nitrogen flow. The evaporation residues from lipid extractions were dissolved into petroleum ether (b.p. 40–60°C; 700 µl). Fatty acids were transmethylated with sodium methoxide (NaOMe; 0.5 M; 250 µl) in dry methanol by boiling at 45°C for 5 min. The mixture was acidified with 15% sodium hydrogen sulphate (NaHSO_4_; 500 µl) and the petroleum ether phase containing the FAMEs as well as FFAs was collected. After centrifugation (10620×***g***, 5 min) the petroleum ether layer was separated into a GC vial and evaporated. The residue was dissolved into hexane (50 µl). The samples (injection volume 1 µl) were analyzed on an Agilent 6890 gas chromatograph combined with Agilent 5973 mass selective detector (MSD). A splitless injection method was used and the injector and MSD interface temperatures were 260°C and 280°C, respectively. The chromatograph was equipped with a FFAP stationary phase column (25 m, ID 200 µm, film thickness 0.33 µm; Agilent 19091F-102) and helium was used as carrier gas. FAMEs were monitored at m/z, 284 (C17:0), 312 (pristanic acid), 326 (phytanic acid), 354 (docosanoic acid, C22:0), 382 (tetracosanoic acid, C24:0), 367 (hexacosanoic acid, C26:0), 91 and 273 (docosahexaenoic acid, C22:6), and FFA (C17:0) at m/z 270.

Serum levels of LH and FSH were measured from four-month-old male mice (*n*=4–6/genotype) by immunofluorometric assays as described earlier (Haavisto et al., 1993; van Casteren et al., 2000). The lower limit of LH detection was 0.75 pg/tube, intra-assay CV <5% at >1 µg/l, and inter-assay CV 7.8%, while the respective levels for FSH were 0.1 µg/l sensitivity and 4.4% and 10% intra- and inter-assay CV, respectively at a level of 4.8 µg/l. Serum testosterone (*n*=5/genotype) was assayed with a radioimmunoassay (Testo-CT2, Cisbio Bioassay, Codolet, France). Functional detection limit was 0.3 nmol/l. Intra- and inter-assay CV 8.9 and 11.6%, respectively.

### Analysis of liver lipids

Lipids were extracted from frozen liver samples (*n*=4–8/group) as described previously (Folch et al., 1957; Siggins et al., 2007). Liver pieces (20–50 mg) were homogenized by use of Thermo Savant FastPrep FP120 instrument and MP Lysing Matrix D tubes (MP Biomedicals, Santa Ana, CA) in 2:1 chloroform:methanol (v/v). The organic phase was washed with 0.9% NaCl solution and dried under nitrogen. The residuals were dissolved in tetraethylammoniumhydroxide (1:28 in 95% ethanol) and hydrolyzed with 0.05 M HCl. Triacylglycerols were measured as glycerol from the end product by use of an enzymatic colorimetric method (GPO-PAP 1488872, Roche Diagnostics, Mannheim, Germany).

### Electron microscopy

For transmission electron microscopy (EM), female mice (*n*=3/genotype) were perfused with 1.0% (w/v) glutaraldehyde in 4% PFA. Liver samples were cut in 2 mm slices, post-fixed in 1.0% (w/v) osmium-tetroxide, dehydrated in acetone and embedded in Epon Embed 812 (Electron Microscopy Science). In mice liver, peroxisomes can be easily recognized by electron microscopy as particles that are surrounded by single membrane, that contain electron dense matrix and a highly specific crystalloid structure named nucleoids.

### Metabolic phenotyping with CLAMS

The effect of high fat containing Western diet TD.88137 (Teklad Lab Animal Diets, Harlan Laboratories, Indianapolis, IN) on metabolic functions was studied in 30 single housed animals (*n*=3–4/group, fed either standard or fat-enriched diet since weaning). At the age of 20 weeks the mice were tested in the Comprehensive Laboratory Animal Monitoring System (CLAMS; Columbus Instruments, Columbus, OH) that consists of eight individual live-in cages that allow automated, non-invasive data collection of several metabolic parameters, e.g. body weight, oxygen consumption, carbon dioxide production, RER, heat production, ambulatory activity and food and water consumption.

During the CLAMS measurements the lights were on between 6:00 and 18:00. The experiment started at 10:00 and continued for 72 h. The period until beginning of the first dark cycle was allowed for acclimatization. The data from the following 60 h (3 dark and 2 light periods) were used for analysis and expressed as mean values per hour during light and dark phase, respectively. The samples were recorded every 30 min. Body weights were determined just before and after testing. Two animals were excluded from the analysis because of losing more than 10% of body weight during the experiment.

Each cage is an indirect open circuit calorimeter that provides measures of oxygen consumption and carbon dioxide production. The system compared oxygen and carbon dioxide gas concentrations by volume at the inlet and outlet ports of the cage chamber through which ambient air flows at a constant rate (0.60 l/min). The difference in concentration between the two ports and the flow information was used to calculate oxygen consumption (ml/kg/h), carbon dioxide production (ml/kg/h) and RER. Heat production (kcal/h) was also estimated with standard formulas using oxygen consumption and the RER. An array of infrared beams (2.5 cm inter-beam distance) surrounded each cage. Ambulatory activity was defined as a movement producing sequential horizontal beam breaks of different beams. Activity was measured continuously and recorded with intervals of one hour as number of beam breaks. A computer monitored continuously a food container on an electronic weigh scale. The consumption was monitored and stored each time a metabolic measure was recorded. Individual water columns containing pressure transducers supplied drinking water to each cage. Drinking reduced pressure in the column and was converted to volume (ml).

### Assessment of peroxisomal function

The peroxisomal function in *Trim37^−/−^* mice was studied in four-month-old old male mice fed with a diet containing clofibrate, a peroxisomal proliferator. Clofibrate [ethyl 2-(4-chlorophenoxy)isobutyrate; Sigma-Aldrich] was supplemented to the mice with their normal chow 0.5% (w/w). One group of mice (*n*=3/genotype) was fed for two weeks with clofibrate and euthanized after treatment. The second group of mice (*n*=3/genotype) was euthanized five days after the two-week clofibrate feeding period. The third group (*n*=3/genotype) of mice consisted of non-treated control animals. The animals were euthanized by cervical dislocation, and serum and liver samples were collected and frozen as described above.

For immunoblotting frozen liver samples (∼50 mg), from clofibrate fed and control mice, were homogenized in 50 mM Tris buffer, pH 8.0 containing 0.5% Nonidet-P40, 10% Glycerol, 0.1 mM EDTA and a cocktail of phosphatase and protease inhibitors. Protein concentrations of the lysates were determined at A595 nm by use of Bio-Rad protein assay chemistry (Bio-Rad Laboratories, Hercules, CA).

Samples for the MFE-1 blots were separated on Mini-PROTEAN TGX Precast Gels (Bio-Rad Laboratories). Immunoblotting was performed using a 1:2000 dilution of MFE-1 antibody raised in rabbit (Palosaari et al., 1990), recognizing the peroxisomal multifunctional enzyme type 1 (MFE-1). Normalization of protein levels was done against β-tubulin (1:10,000 mouse anti-β-tubulin, Sigma-Aldrich). Secondary antibodies used were 680 Donkey anti-mouse (Molecular Probes, Eugene, OR) and IRDye 800CW Goat anti-Rabbit IgG (LI-COR Biosciences, Lincoln, NE). Quantification of MFE-1 protein from the scanned blots was performed with the LI-COR Odyssey Western Blot Detection System.

ADRP levels were immunoblotted from liver samples (40 µg of protein per lane, even loading verified by Ponceau A staining of the blotted membrane) by using a 1:3000 dilution of anti-ADRP antibodies (Progen Biotechnik GmbH, Heidelberg, Germany) followed by horseradish peroxidase-conjugated donkey anti-guinea pig IgG antibodies (Jackson ImmunoResearch Laboratories Inc., West Grove, PA). The signal was visualized using enhanced chemiluminescence western blotting detection reagent (ECL) (Amersham Biosciences, Amersham, UK). Densitometric analysis of the blots was done with ImageJ software (http://imagej.nih.gov/ij/index.html).

### Statistical analysis

Statistical differences between two groups were tested by *t*-test. All data are expressed as mean values with standard deviations (s.d.).
